# Alcohol and Drug Facilitated Sexual Violence: Using the World Café Method for a Multi-Stakeholder Collaborative Approach to Prevention Development

**DOI:** 10.1177/10778012251379421

**Published:** 2025-09-19

**Authors:** Kirsty Forsdike, Jessica Ison, Elena Wilson, Jacqui Theobald, Mette Hotker, Alex Donaldson, Hannah Korsmeyer, Leesa Hooker

**Affiliations:** 1 La Trobe Rural Health School, 2080 La Trobe University, Bendigo, Victoria, Australia; 2 Centre Against Sexual Assault Central Victoria, Bendigo, Australia; 3 Centre for Sport and Social Impact, 2080 La Trobe University, Bundoora, Victoria, Australia; 4 Monash University, Melbourne, Australia

**Keywords:** sexual violence, alcohol, drug, prevention, World Café

## Abstract

This article presents a study exploring the prevention of alcohol and drug (AOD)-facilitated sexual violence. A participatory action research/appreciative inquiry method, World Café Forum, was used to take a multi-stakeholder approach to explore prevention initiatives. Thirty-two individuals from 14 stakeholder organizations attended. Analysis established five recurring themes, overlayed by power imbalances: education and training; policy-led initiatives; holding people accountable; social information campaigns; and cultural change. Responsibility for addressing the issue is contested. The greatest opportunity to address AOD-facilitated sexual violence lies with organizations, with a focus on restorative justice. Policy frameworks and place-based initiatives are required.

## Introduction

Sexual violence is a global health issue mostly affecting women (World Health Organisation, 2021). In Australia, 23% of women will experience sexual violence across their lifetime, compared to 8% of men (Australian Bureau of Statistics, 2021). Sexual violence is reported to be higher in rural than urban areas, although prevalence is still relatively unknown, particularly for young women (Australian Bureau of Statistics, 2017; Hooker et al., 2019).

The World Health Organisation defines sexual violence as “any sexual act, attempt to obtain a sexual act, unwanted sexual comments or advances, or acts to traffic, or otherwise directed, against a person's sexuality using coercion, by any person regardless of their relationship to the victim” (World Health Organisation, 2013). It has significant psychological and physical health impacts for women, including posttraumatic stress disorder (PTSD) and gastrointestinal issues (Dworkin, 2020; Tarzia et al., 2017; World Health Organisation, 2014). Sexual violence is most frequently experienced by women and LGBTQ+ people (Ison et al., 2025a), and those who face intersecting forms of inequality can experience higher rates of sexual violence. For example, women with disabilities or trans women of color have experienced higher rates of sexual violence (Australian Institute of Health and Welfare, 2024; Hindes et al., 2025; Ledingham et al., 2022).

Increasingly, it is being recognized that alcohol and drugs (AOD) are used to facilitate sexual violence. Alcohol and other drug facilitated sexual violence includes what is often colloquially known as “drink spiking” (Ison et al., 2024). Perpetration can be opportunistic, such as where the perpetrator takes advantage of a person who is intoxicated, and/or proactive, such as intentionally administering a substance to incapacitate a person (Gee et al., 2006). The victim may consume AOD voluntarily or be unaware that they have been administered them (Caluzzi et al., 2025). Alcohol and other drug facilitated sexual violence can also include the perpetrator encouraging the victim to become further intoxicated (Ison et al., 2025b). Available evidence indicates the most likely substance used by perpetrators is alcohol, but they may also use other sedative substances such as flunitrazepam (Rohypnol) or other benzodiazepines and gamma-hydroxybutyrate (GHB) (Anderson et al., 2017; Recalde-Esnoz et al., 2024; Wolitzky-Taylor et al., 2011).

Responses to AOD-facilitated sexual violence have often been piecemeal. The service system response often lacks continuity of care, and while staff may be passionate and caring, they are often overworked and have limited knowledge or training on AOD-facilitated sexual violence (Ison et al., 2025c). There have been some attempts at programs to address AOD-facilitated sexual violence, though there have been limited rigorous evaluations. These interventions have tended to focus on bars and clubs, particularly through training bar staff as bystanders (Davis et al., 2024), including a resource for bar staff that we designed for the larger project that this study is part of (detailed below) (Hooker et al., 2024). Interventions also include “solutions” to drink spiking, such as a scrunchie to cover one's drink, or nail polish to test whether there are substances in your drink. These supposed solutions often place the onus on women to keep themselves safe through feminized products, which have troubling victim-blaming undertones (Clinnick et al., 2024).

Beyond such examples, the vast majority of interventions are focused on alcohol consumption in US college settings. While they may have some specific focus on AOD-facilitated sexual violence, they are generally concerned with minimizing the intake of alcohol. Very few interventions are focused on prevention (Hooker et al., 2020) or on response that goes beyond individuals to consider how to change broader sociocultural contexts (Dworkin & Weaver, 2021).

### Study Context

There has been growing interest in and reporting on “drink spiking” in the media. In 2021, the media highlighted “drink spiking” as an issue in a regional town in Victoria (Cunningham & Koob, 2021; Lawrence & Findlay, 2021). Some young women came forward to talk to journalists about their experiences of drink spiking in a local club and the subsequent negative interactions they had with health and justice services. These media reports also indicated that drink spiking is an issue in rural communities broadly and that victims face significant barriers when seeking assistance through health and justice services. As with sexual violence broadly, increased media reporting does not necessarily mean there is an increased prevalence, but rather that people may feel empowered to come forward (Clinnick et al., 2024). The stories of the young women in the media reports inspired the research team to conduct a study focused on regional and rural experiences of AOD-facilitated sexual violence. To date, little research has been conducted on rural and remote communities’ experiences of AOD-facilitated sexual violence. However, research has shown that rural and regional Australia have distinct issues relating to sexual violence compared to urban areas, such as dominance of rural hegemonic masculinity and sexual violence revictimization (Corbett et al., 2023; Saunders & Easteal Am, 2013). The study underpinning this paper explored how a regional community could respond to, and ultimately prevent, AOD-facilitated sexual violence (Hooker et al., 2024). This article reports the findings from one part of the study: the use of a multi-stakeholder participatory action method known as a World Café Forum.

## Methods

The World Café Forum is a collaborative qualitative method used to foster “constructive dialogue, accessing collective intelligence, and creating innovative possibilities for action” (Brown, 2005). It derives from participatory action research and appreciative inquiry methods that aim to guide a large group of diverse stakeholders toward solutions (Aldred, 2011). It has been used in community development (Aldred, 2011) and where interprofessional collaboration is required, for example, in healthcare and violence against women (Breitbach et al., 2017; Forsdike & Fullagar, 2021). The method brings together multiple small conversational groups to build one collective conversation of different perspectives (Brown, 2005). To build a collective conversation, participants are required to move between groups and discussion topics, so that previous conversations are built upon and include new perspectives for action (Brown, 2005).

A World Café forum was held in 2022 in a regional town in Victoria, Australia, bringing together multiple stakeholders to consider AOD-facilitated sexual violence and how it could be prevented in the region. The forum was conducted over the course of a full day and consisted of two parts. The first half of the day included presentations by members of the research team on sexual violence and AOD-facilitated sexual violence, as well as evidence of the issue in the local community. The presentations were used to engage participants and disseminate existing knowledge about the phenomena and focus on the local region. The second half of the day, the results of which this article reports, incorporated World Café method discussion groups informed by the information provided earlier in the day. The project received ethical approval from the first author's institution (approval reference: HEC22254).

One of the key features of the World Café method is that participants rotate around the tables every 20–30 min (Fouché & Light, 2011). A host remains at their designated table to support discussion, continuity, and the development of ideas arising from previous conversations (Brown, 2005). Such varied perspectives on issues and the ideas developed are unlikely without facilitated interaction between a broad and diverse range of participants (Brown, 2005).

There are seven principles in the method's application which were followed on the day (see Table 1).

**Table 1.** Principles and Application of World Café Method.

Principle (Brown, 2005)	Application for This Project
Setting the context	Three components to setting the context Inviting a variety of key stakeholders covering policy, health, youth, alcohol and drug and sexual violence services, police, and multidisciplinary researchers who could inform discussions about AOD-facilitated sexual violence in the local community Delivering presentations that shared knowledge and current evidence about AOD-facilitated sexual violence and its prevention Developing questions to orient discussion groups arising from the presentations
Creating a hospitable space to open collective conversations by encouraging trust and respect for differences	Establishing a relaxed and friendly space with natural lighting, and the room set up with small tables of four to six people with space to move about, materials for engagement and recording perspectives (pens, large sheets of paper, sticky notes), a break-out space with food and drink to support informal conversations
Exploring questions that matter	Establishing questions for discussion groups that drew on the presentations
Encouraging everyone's contribution	Providing a host for each table to support discussions and their continuation between groups.
Cross-pollination and connective diverse perspectives	Rotating diverse participants across tables every 20–30 min
Listening for patterns, insights, and deeper questions	Host remaining at their designated table to support continuity and develop ideas arising from previous conversations
Harvesting and sharing collective discoveries	Using various tools and materials (pens, paper, objects) to record perspectives and potential for action

Firstly, two questions informed by the earlier presentations were posed to the discussion groups to introduce AOD-facilitated sexual violence and establish a collective understanding of what it is in the region and how it is currently responded to by the organizations participants were representing (Brown, 2005).

Secondly, the key question then posed to the discussion groups, and which we present in the results below, was “What can we do?” Records of participants' ideas were pinned to the walls to enable participants to reflect upon the discussions in other groups (Fouché & Light, 2011). Research team members took photos of these records for analysis.

### Analysis

Analysis was informed by the socioecological model. The model was originally developed by Bronfenbrenner to reflect the relational and multiple forces that shape experience across individual, relationship, community, and sociocultural levels (Bronfenbrenner, 1977). It was further developed by Heise to provide a framework for understanding violence against women (Heise, 1998). Heise argued that we need to understand the different levels and their integration to improve responses to a complex issue (Heise, 1998). The model has since been adapted to consider imbalances of power within and between the socioecological levels (Forsdike & Giles, 2024).

The records were transcribed by co-author Jessica Ison and thematically analyzed by co-authors Kirsty Forsdike and Elena Wilson (Braun & Clarke, 2022), with co-authors Jessica Ison and Kirsty Forsdike meeting to finalize themes once co-author Jessica Ison had reviewed the initial themes developed.

## Results

Thirty-two stakeholders from 14 different organizations attended the World Café Forum, with an additional seven facilitators attending from the project team. Of the 32 stakeholder attendees, 78% (*n* = 25) were women. The range of organizations or services from which they derived is presented in Table 2, and included specialist violence prevention and response services, health services, police and justice representatives, students, and student services.

**Table 2.** World Café Forum Participants.

Stakeholder Type	No. People Attending
Specialist violence prevention and response service	9
Police and justice	6
Health service	5
Multicultural service	3
Higher education students	4
Higher education student services	2
Policy	2
Media	1

**Table 3.** Themes.

Socioecological Model Level	Relevant Theme	Component of Relevant Theme
Individual	Training and education	Training: bystanders and those working in security at hospitality venues; individual safe drug taking
Relational	Training and education	Education: parents to children; through existing training programs aimed at respectful relationships and consent training
Organizational	Training and education	Training: through healthcare, police, and with venue licensing; place-based training
	Policy-led initiatives	Hospitality Health Services Tax
	Holding people accountable	Justice/police response Victim Taking responsibility
	Social information campaigns	
Sociocultural	Policy-led initiatives	Drug and alcohol policies
	Cultural change	

We generated five recurring themes through analysis: (a) training and education, (b) policy-led initiatives, (c) holding people accountable, (d) social information campaigns, and (e) cultural change. When aligning these with the socioecological model (Table 3), it is clear that forum participants considered the organizational level to be the area of greatest opportunity for initiatives, followed by the sociocultural level. The individual and relational levels of the model were not identified as providing many pathways for addressing AOD-facilitated sexual violence in the community.

### Education and Training

Unsurprisingly, education and training were dominant themes in discussions. Education refers to building understanding around AOD-facilitated sexual violence, while training refers to skill capacity building to respond to AOD-facilitated sexual violence. Some of the educational measures proposed addressed how people relate with each other, aligning with the relational level of the socioecological model. Here, participants discussed parenting education, engaging with the parent–child relationship to address AOD-facilitated sexual violence. Participants also referred to embedding such education within existing education programs, such as Respectful Relationships and sexual consent: “Comprehensive sexual consent education embedded into all educational institutions, i.e., what consent looks like and the nuances around this when using AOD.”

There was a focus by participants on peer education so that boys would educate boys in understanding and addressing AOD-facilitated sexual violence. Education of AOD-facilitated sexual violence also sits within the organizational level of the socioecological model, whereby it should form part of lifelong learning throughout early years education, primary school, secondary, and tertiary education.

Skills development within organizations such as police and healthcare, and places such as the workplace, at music events, sports clubs, and LGBTQIA+ events were also identified by participants. At the individual level, training was identified as essential for those working in hospitality security specifically (including developing the skills in “identifying and acting on AOD-facilitated sexual violence”), bystander training and safe substance use training for individuals.

### Policy-Led Initiatives

Participants identified an absence of policy frameworks and initiatives in relation to AOD-facilitated sexual violence and argued that this was required at the organizational level and across various domains, including hospitality, health systems, and taxation. Discussions among participants produced some specific suggestions for initiatives such as “bringing alcohol service in line with food service (quality control, etc.)” and “align planning laws with hospitality, e.g., co-located supports for AOD-facilitated sexual violence.”

The latter initiative of a co-located support referred to venues being close to support services. Participants discussed co-location at length, detailing planning applications for hospitality venues such as pubs requiring recognition of where there were support services or requiring new venues to co-locate with support services. There were several participants in attendance who worked in specialist violence prevention and response, and women's services, and they raised that alcohol and other drug services should be integrated with family violence, sexual violence, and mental health services at both the policy and service system levels.

Threaded throughout these discussions was the need for culturally specific responses to alcohol and drug issues. Tax policy initiatives proposed related to a “big alcohol tax” and the profits from tax being “used in harm minimization.” The remaining subthemes within policy-led initiatives align more with the sociocultural level of the socioecological model. This incorporated suggestions such as decriminalizing illicit drugs, normalizing safe substance use, limiting or regulating alcohol, and reporting guidelines for the media.

### Holding People Accountable

The discussions were particularly forceful when considering the need to hold people accountable. At the organizational level, participants were most concerned with holding licensed venues accountable or requiring them to take some responsibility for preventing AOD-facilitated sexual violence. Harsher enforcement of penalties for venues where AOD-facilitated sexual violence takes place was proposed alongside an independent body (“watch dog”) to hold venues accountable, which includes “access to CCTV—and allow it to be viewed openly.” But more often, the participants discussed the need for initiatives that were led by or took place in licensed venues; for example, mandated AOD-facilitated sexual violence programs for licensed venues and safety officers located at venues. Another specific initiative suggested bringing licensed venues together “to create a shared onus of responsibility/plan.” In relation to perpetrators, at the individual level, participants considered the need to hold “abusers accountable within systems that actually rehabilitate” and ensuring that there are sufficient resources “to speed up processing perpetrators of AOD-facilitated sexual violence.” Linked to this was the focus on victim-led responses, for example, local restorative justice or “alternative pathways for justice for victim survivors.”

### Social Information Campaigns

Participants specified initiatives for their local region when discussing social information campaigns. While general ideas were generated and proposed for public health campaigns around male behaviors, or awareness-raising campaigns in venues and public toilets, taxis, and social media, the rural focus of the project generated interesting locations for such campaigns. The need to focus on male behaviors was emphasized rather than what was seen as the current focus on women's behaviors. For example, participants reported on an art exhibition they had seen in the news that was held at the United Nations Headquarters in New York City. The exhibition showcased the variety of clothing women who have been raped were wearing to dispel long-held rape myths. Participants attending the World Café Forum wanted campaigns on the back of toilet doors that directly questioned men: “have you used substances to manipulate some into sex?”

The region where the World Café was conducted has a well-known recreation area [Rosalind Park] where major events are held, and participants suggested that campaigns could be linked to popular events in this location. They suggested that including safe space tents should be required when holding an event. Similarly, participants suggested encouraging the city council “to focus on this as part of community safety week.”

### Cultural Change

Cultural change, as part of the sociocultural level, was recognized across the discussion groups as difficult but necessary to address AOD-facilitated sexual violence. Cultural change was argued to be needed around gender inequality. It was well recognized by the specialist and women's health services in the room that gender inequality is associated with sexual violence. In particular, participants highlighted male entitlement and control with the need to “address male entitlement in relation to respect for women,” “change ideas of male ownership/control,” and “believing women.” Participants also reflected on shifting narratives, for example, “shift the narrative” in relation to cultural attitudes around drugs and alcohol, “changing alcohol culture,” and “shifting student culture so people can speak out.” These narrative shifts identify two concepts: the Australian collective attitude toward AOD, and the ability of an individual within the culture to speak up, particularly in rural and regional areas. One participant group specifically noted that there was a “Reluctance among men to dob mates in and this is a bigger challenge in rural towns where men can then be ostracized from their community.”

### Power

In recognition of the development of the socioecological model and its adaptation to consider imbalances of power within and between the socioecological levels, we were sensitive to this concept as we considered the themes detailed above (Forsdike & Giles, 2024).

Throughout the forum, power was a recurring topic discussed overtly in terms of who holds power over victims of AOD-facilitated sexual violence. For example, participants discussed how licensed venues hold power over their patrons, particularly over women who frequent them and are subjected to AOD-facilitated sexual violence. Alongside discussion of power imbalances, participants drew out some of the more covert power imbalances. In particular, participants talked about how the broader patriarchal cultural contexts see men holding power over women, which is at times heightened in rural communities and for minorities. We reflect on this more in the discussion below.

## Discussion

The World Café method brings together people from a variety of perspectives and backgrounds to discuss an issue of importance. Our forum produced important findings on how to respond to and prevent AOD-facilitated sexual violence, particularly in regional and rural communities. Participants were candid about how AOD-facilitated sexual violence is a topic that can be challenging to tackle. Even those from specialist services can struggle to integrate the two issues of (a) alcohol and other drugs and (b) sexual violence. Those working in AOD-facilitated sexual violence need support for greater understanding of the term and to be able to tackle it from a cohesive perspective rather than from either an AOD or a sexual violence perspective.

As noted in the results, power was a recurring topic in terms of who holds power, for example, licensed venues holding power over women patrons. Yet, venues are unlikely to be expected to deal with or be held accountable for AOD-facilitated sexual violence that occurs at their venue. An unwillingness to assume responsibility is reflected in broader gender-based violence. For example, organizations such as universities or workplaces are often reluctant to acknowledge, let alone take responsibility for, preventing and responding to sexual harassment. As a result, victims struggle to find integrated service systems and are often forced to engage with multiple services when seeking support, resulting in poor continuity of care (García-Moreno et al., 2015). The issue of who is responsible for preventing, responding to, and supporting victims of AOD-facilitated sexual violence needs further exploration, discussion, and recognition, given the number of stakeholders involved (Ison et al., 2025c).

With regard to covert power imbalances, there are often troubling power imbalances that victim-survivors of sexual violence face at all levels of the socioecological model (Tarzia, 2020). This was identified through Australia's patriarchal cultural context, recognized as particularly dominant in rural communities and for minorities. This understanding of sexual violence allowed participants to consider how to address AOD-facilitated sexual violence beyond just standard approaches of behavioral change to considering how to prevent sexual violence through broader cultural change, often referred to as primary prevention (Hooker et al., 2020).

One suggestion for addressing power imbalances was to implement transformative justice responses to victim-survivors. This reflects the demographics of the participants, with many working in the gender-based violence sector and in feminist advocacy, which has engaged in transformative justice work (Rasmussen, 2022). Transformative justice, as used in feminist advocacy, comes from anticarceral approaches, particularly those led by Indigenous people and people of color (Davis, 2019). Approaching sexual violence perpetration from a noncarceral perspective is something being taken up—though at times removed from these decolonial and antiracist approaches—by universities and other institutions (McMahon et al., 2024). To date, transformative justice for victim-survivors of AOD-facilitated sexual violence has been underexplored and offers a possible new avenue of research and advocacy. Restorative justice processes could also be an opportunity for perpetrators of AOD-facilitated sexual violence to recognize their behaviors and their impact. Transformative justice response broadly highlights the investment from those working with victim-survivors to considering alternative approaches outside of the current criminal-legal approach. Participants advocated for such an approach to focus on restoring power to victim-survivors.

Integrated prevention and response systems that are place-specific while also addressing both specific initiatives and broader issues, such as gender inequality, are key across all ages, stages, and places. Participants talked about needing responses to AOD-facilitated sexual violence that were culturally specific, particularly to the regional and rural context. Such an interconnected prevention approach system must consider the nuanced and place-specific, addressing both specific initiatives and broader issues such as gender inequality. It is crucial to develop strategies that are adaptable to the unique needs of different communities to be effective.

Given that participants were predominantly from regional areas, it is unsurprising that they advocated for location-specific responses relevant to their local community. They suggested embedding responses to and preventing AOD-facilitated sexual violence at key local events as well as having them embedded in community hubs, co-located service spaces. Community responses to sexual violence have been identified as an important approach for prevention (Hooker et al., 2021). However, to date, community-based responses have been underresourced with limited evaluations (DeGue et al., 2016). Existing programs tend to focus on troubling victim-blaming approaches such as drink cover (Clinnick et al., 2024) or training bar staff (Davis et al., 2024; Hooker et al., 2024). Given that drink spiking often garners significant media attention (Clinnick et al., 2024), including in the region where this study took place, it offers an opportunity for large-scale community engagement in prevention.

One of the limitations of the World Café Forum was the voices that were missing in the room. Despite invitations, no one from hospitality attended. Given this is a prominent location for AOD-facilitated sexual violence, it was disappointing that those working in hospitality locally did not attend, but it is perhaps reflective of their unwillingness to see a role in addressing the issue. The other limitation of a World Café Forum is the potential imbalance of power in the room. This can lead to dominant voices, reduced opportunity for dissenting voices, and the potential for certain voices to be silenced. For example, those facilitating discussions were aware that older and more experienced people in the work tended to dominate some of the conversations. This meant that facilitators based at each group discussion needed to deftly negotiate the voices, but there could have been some voices lost in the process.

## Conclusion

This article reports findings from a World Café forum that brought together stakeholders from a variety of perspectives and backgrounds to discuss AOD-facilitated sexual violence. The aim of the forum was to produce conditions whereby participants could share knowledge and views on what ought to be done to respond to the issue in their regional area. The findings from discussions have implications for public health. Reflecting a shared view that sexual violence signals deeply embedded gendered power imbalances in society, participants overwhelmingly saw that responding to and preventing AOD-facilitated sexual violence should be chiefly undertaken at the organizational and sociocultural level. A dearth of policy frameworks and initiatives responding to the problem was identified, and it was evident there was a lack of agreement concerning who should assume responsibility for tackling the problem, alongside concern that powerful stakeholders such as licensed venues were rarely held to account. A range of measures were suggested, with a particular focus on the implementation of restorative justice approaches—reflecting the view that social policy and service delivery should restore power to victim-survivors. The importance of community-based responses relevant to local communities was also emphasized alongside targeting the behavior of men (not women)—a perspective that locates responsibility for AOD-facilitated sexual violence with perpetrators.
